# A novel parameter derived from post-processing procedure of dual energy CT for identification of gout

**DOI:** 10.1038/s41598-021-01100-0

**Published:** 2021-11-03

**Authors:** Chunlin Xiang, Hongyan Zhang, Gang Wu

**Affiliations:** 1 Department of Radiology, Tongji Hospital, Tongji Medical College, Huazhong University of Science and Technology, Wuhan, China; 2 Department of Obstetrics and Gynecology, Tongji Hospital, Tongji Medical College, Huazhong University of Science and Technology, Wuhan, China

**Keywords:** Musculoskeletal abnormalities, Osteoarthritis, Muscle

## Abstract

ROI analysis is frequently used for obtaining acid content on rapid-kV-switching dual energy CT (DECT), providing inadequate accuracy. A new parameter derived from post-processing procedure, maximum lower limit with stain visible (MLLSV), was used by us to diagnose gout. 30 gout patients and 20 healthy volunteers were analyzed by using MLLSV. MLLSV was defined as the maximum lower limit of display window allowing only one stained site visible. Radiologists were asked to continuously increase the lower limit of display window of uric acid to decrease number of stained sites until the last stained site disappeared. MLLSV obtained by this way was compared between gout patients and volunteers. Receiver operating characteristic (ROC) curve was used to determine the performance. MLLSV of gout patients was significantly higher than that of volunteers (1373.3 ± 23.0 mg/cm^3^ vs. 1315.4 ± 20.7 mg/cm^3^, *p* = 0.000). The area under ROC curve of MLLSV was 0.993 in identifying gout. When using the optimal cutoff of 1342 mg/cm^3^, the sensitivity and specificity of MLLSV in identification of gout were 96.7% and 95% respectively. MLLSV derived from post-processing procedure of DECT is useful in discriminating gout patients from healthy people.

## Introduction

Gout is a crystalloid joint disease more common in adult males, with monosodium urate crystals deposited in joints and periarticular soft tissues^1^. Chronic hyperuricemia predisposes to gouty arthritis^2^. The diagnosis of gout is mainly based on clinical manifestations and laboratory tests. Elevated level of uric acid in the serum correlates closely with gout. However, some patients have asymptomatic hyperuricemia, which is not necessarily to develop into gout^3^. Joint aspiration is considered as the reference standard for the diagnosis of gout^4^. However, detection of uric acid crystals using aspiration may become difficult when synovial fluid is insufficient. Invasive joint aspiration increases the risk of bleeding and infection. In addition, aspiration is also limited in small joints.

Dual energy computed tomography (DECT) is reported as a valuable method for the diagnosis of gout^5–7^. DECT was included in the 2015 American College of Rheumatology/European League Against Rheumatism (ACR /EULAR) Collaborative Initiative Classification Criteria for Gout^8^. In clinical practice, DECT for gout diagnosis mainly includes dual-source DECT and rapid-kV-switching DECT^9,10^. In recent years, most of studies used dual-source CT for the diagnosis of gout^11–14^. It is well established dual-source CT performs well at visualizing uric acid crystal. However, dual source CT is a very expensive system, which was unavailable for some centers, especially in developing countries. Compared to dual source CT, rapid-kV-switching DECT is the more common system in clinical practice. However, there were few publications using rapid-kV-switching DECT for assessing gout. Among rapid-kV-switching DECT post-processing methods, region of interest (ROI) analysis is most frequently used for estimating uric acid content of patients^15^. However, this method provides inadequate accuracy in identification of gout, due to the overlap of uric acid content between diseased and normal tissues.

Gemstone energy spectrum CT adopts dual energy CT imaging with 80 kV and 140 kV rapid switching as the core to achieve material decomposition, which can be used to determine the material content of human peripheral joints^16^. Through the material decomposition technology, Gemstone energy spectrum CT can generate the pairing map of uric acid and hydroxyapatite for measuring the content of uric acid^15^. On the uric acid (hydroxyapatite) map, the stained method helps to localize the areas with higher content of uric acid. Number of stained sites varies with the lower limit of display window of uric acid. The maximum lower limit with stain visible (MLLSV) allows for only one stained site to be visible. If using a higher lower limit, this stained site would disappear. MLLSV of joint can be easily obtained by continuously increasing the lower limit of display window of uric acid, making stained sites number to decrease, until the last one disappeared. However, there is no publication reporting using MLLSV for identification of gout to the best of author’s knowledge. The current study thus aimed to investigate the feasibility of MLLSV in discriminating gout patients from healthy people.

## Methods

All methods were carried out in accordance with relevant guidelines and regulations.

### Study participants

**Figure 1:**
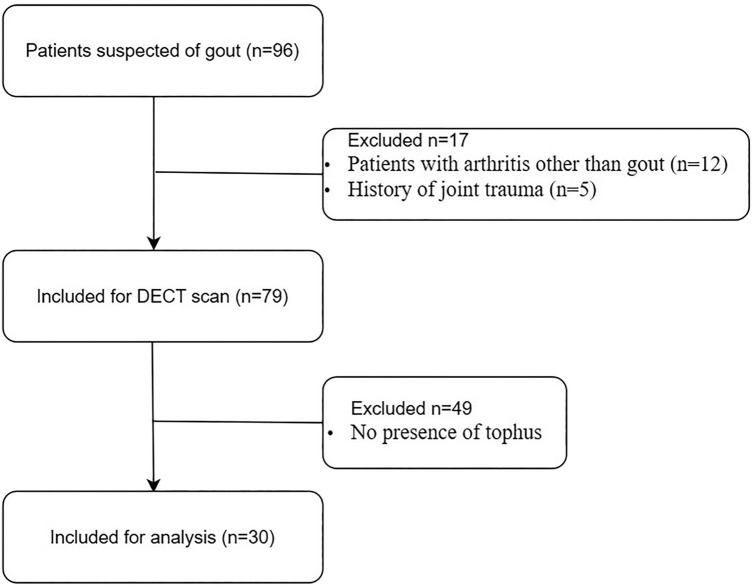
Flow chart indicating gout patients recruitment, inclusion, and exclusion.

**Figure 2:**
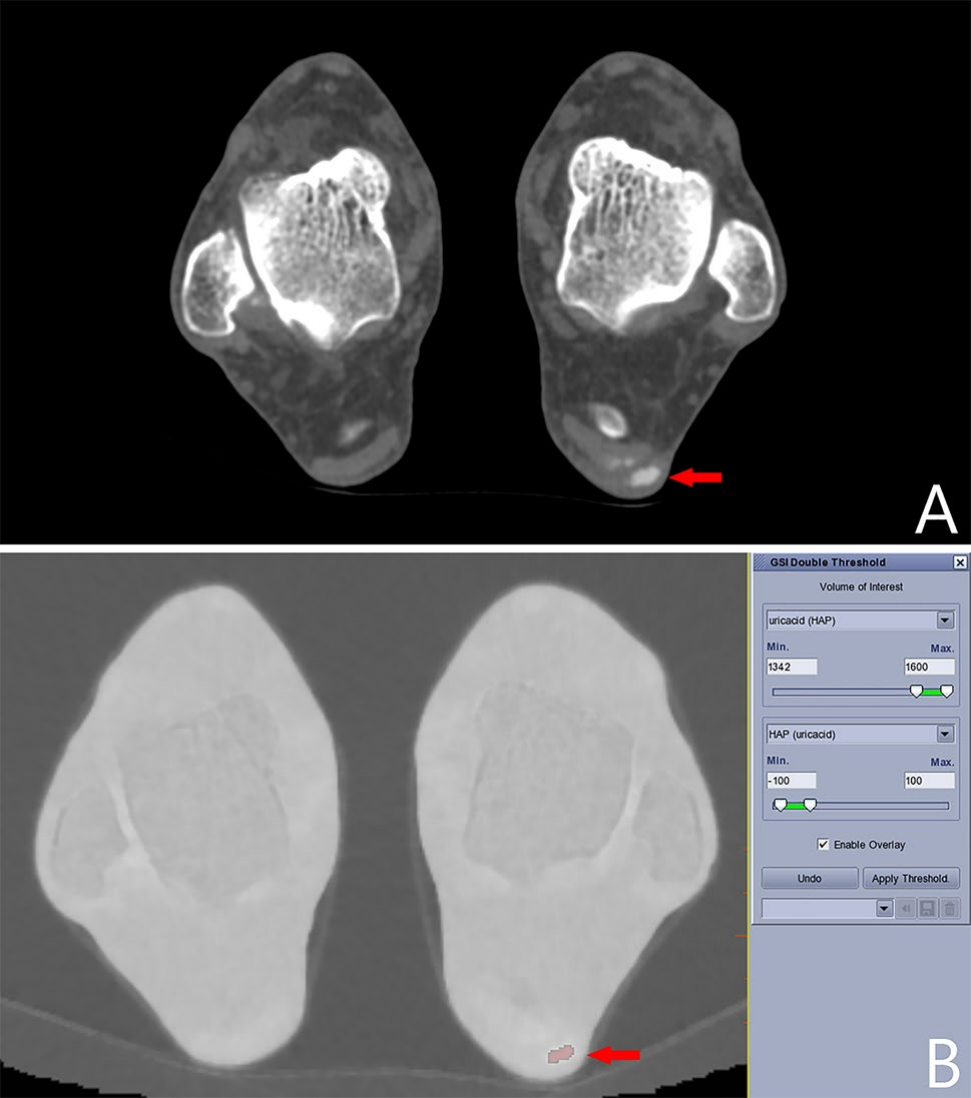
A 56 years old male with acute pain of left foot and ankle joint, serum uric acid 517.6 μmol/L. On the gray scale image (**A**), a tophus (arrow) can be seen in the achilles tendon area behind calcaneus. On the uric acid (hydroxyapatite) map (**B**), the tophus was stained red with the threshold of 1342 mg/cm^3^.

This prospective study was approved by the Ethics Committee of Tongji Hospital of Tongji Medical College of Huazhong University of Science and Technology. All participants were informed and signed informed consent. From January 2019 to December 2019, the study performed DECT scans for patients suspected of gout of the ankle and foot joint. Exclusion criteria were as follows: (1) arthritis other than gout; (2) history of joint trauma; (3) absence of high-density tophus in CT images. A total of 30 patients with tophus were finally analyzed (see Fig. 1), who conformed to clinical diagnosis of gout according to ACR /EULAR Criteria^8^. The tophus was a high-density nodule on conventional grey-scale CT images (Fig. 2A). We recruited 20 healthy volunteers for comparison. The volunteers had no history of peripheral joint diseases or related symptoms, and had normal laboratory findings including serum level of uric acid.

### CT scan

Dual energy CT (Discovery CT750 HD, GE Healthcare) was used in the study. Bilateral ankle joints and foot joints were scanned for gout patients and healthy volunteers. The scanning scope includes all joints and periarticular soft tissues of bilateral ankle joints, bilateral tarsal joints, and bilateral metatarsophalangeal (MTP) joints. The scanning parameters were as follows: tube voltage (80/140kVp rapid switching), tube current 360 mA, pitch 0.984:1, detector collimation 64 × 0.625, gantry rotation speed 0.6 s, layer thickness 5 mm, layer spacing 5 mm, reconstruction layer thickness and layer spacing 0.625 mm, reconstruction mono energy 70 keV.

### Data analysis

**Figure 3:**
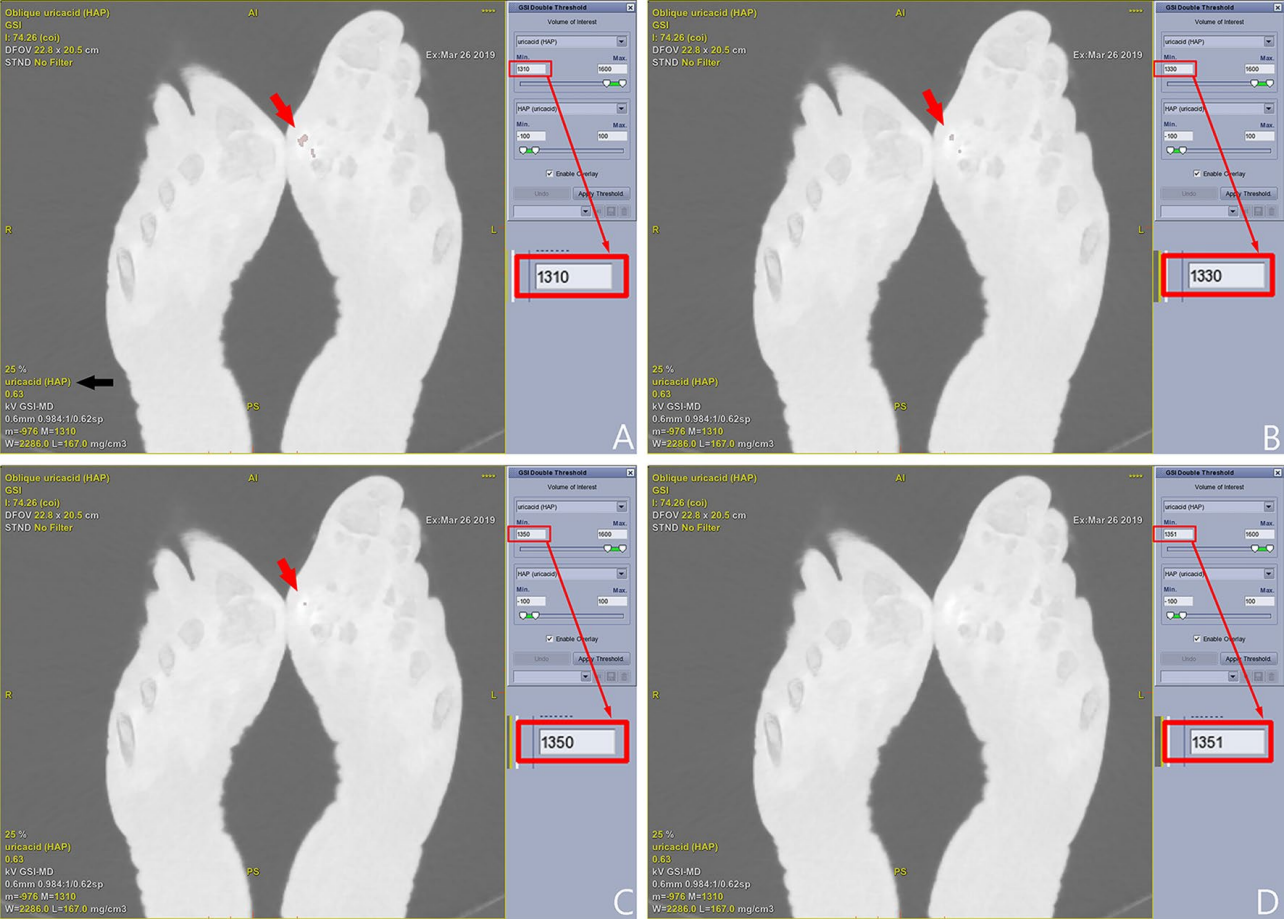
An example of obtaining MLLSV. The detection threshold of HAP (uricacid) map is set as − 100 ~ 100 mg/cm^3^. The lower limit of detection threshold (mg/cm^3^) is adjusted on the uricacid (HAP) map. Multiple stained sites [red arrow in (**A**)] were shown in the left first MTP joint with lower limit of 1310 mg/cm^3^ (red rectangle) of the uricacid (HAP) map [black arrow in (**A**)]. The number of stained sites [arrow in (**B**)] decreased at lower limit of 1330 mg/cm^3^ [red rectangle in (**B**)]. Only one stained site [arrow in (**C**)] is visible with the lower limit of 1350 mg/cm^3^ [red rectangle in (**C**)]. The last stained site is not seen with lower limit of 1351 mg/cm^3^ [red rectangle in (**D**)]. MLLSV is thus 1350 mg/cm^3^ for this case. MLLSV = maximum lower limit with stain visible. HAP (uricacid) = hydroxyapatite (uric acid), uricacid (HAP) = uric acid (hydroxyapatite).

The source data of DECT of ankle and foot was transferred to GE AW4.72 workstation for processing. Two radiologists (C.L.X. and G.W., with 8 and 10 years of experience, respectively) performed the data analysis separately. Gemstone Spectral Imaging (GSI) Volume Viewer was used to generate uric acid (hydroxyapatite) and hydroxyapatite (uric acid) maps. The detection threshold of hydroxyapatite (uric acid) map was set as − 100 ~ 100 mg/cm^3^. With the increase of lower limit of uric acid crystal detection window, the number of stained sites decreased gradually, until only one stained site could be seen. If using a higher lower limit, the last stained site would be no longer visible. By continuously changing the lower limit of detection window of uric acid, MLLSV could be easily obtained (Fig. 3). Two radiologists separately determined the MLLSV of each case.

MLLSV was separately analyzed for the following joints: ankle joint, tarsal joint, and first MTP joint. MLLSV of joint corresponds with the lower limit allowing for only one stained site visible in joint.

One month later, MLLSV was measured for the second time by one of the two radiologists (C.L.X. with 8 years of experience).

ROI analysis in uric acid (hydroxyapatite) map was separately performed for the following six joints: right ankle joint, left ankle joint, right tarsal joint, left tarsal joint, right first MTP joint and left first MTP joint. Oval ROI was consecutively put on multiple cross sections in order to cover the whole joint. ROI was asked to be as large as possible, but should not cover skin or bone. The mean value of each ROI was recorded. Values from multiple slices were averaged to obtain the value of joint. Values from left and right sides were averaged on joint level. Values from six joints were averaged to obtain the value of patient. One month later, ROI analysis was performed for the second time by one of the two radiologists (C.L.X. with 8 years of experience).

### Statistical analysis

All statistical analysis was performed using SPSS software (version 22.0, IBM statistics, USA). The continuous variables were presented as mean ± standard deviation. Intra-class correlation coefficient (ICC) was calculated to determine the inter-reader and intra-reader reproducibility in measuring uric acid content with ROI and MLLSV. The independent sample t test was used for data that conforms to the normal distribution, and the Mann–Whitney U test was used for data that does not conform to the normal distribution. Receiver operating characteristic (ROC) curve was used for MLLSV and uric acid content for further analysis. The Z test was used for the comparison of area under ROC curve (AUC). *p* < 0.05 was considered to be statistically significant.

## Results

### Participants information

There were 29 males and 1 female in gout group, with an average age of 45 ± 16 years, with age range 25–83 years. The average serum uric acid level was 522.1 ± 117.6 μmol/L (range 330–830 μmol/L) for gout patients. The median duration of gout was 5 years (range 0.1–20 years). 17 patients (17/30, 56.7%) received treatment, including medication and diet control.

There were 19 males and 1 female in volunteer group, with an average age of 48 ± 14 years, with age range 22–78 years. The average serum uric acid level was 258.4 ± 42.9 μmol/L (range 165–311 μmol/L) for volunteers. In our laboratory, the normal reference value of serum uric acid ranged from 143 to 339 μmol/L. There was no significant difference in age or gender between gout group and volunteer group (*p* = 0.462; *p* = 0.771, respectively).

In the gout group, 60 ankle joints, 60 tarsal joints and 60 first MTP joints were included in analysis of joint MLLSV and uric acid content. In the volunteer group, 40 ankle joints, 40 tarsal joints and 40 first MTP joints were included in analysis of joint MLLSV and uric acid content.

MLLSV was successfully obtained for all patients and volunteers. Inter-reader ICC for MLLSV was 1.0, either in people level or joint level. Intra-reader ICC for MLLSV was 1.0, either in people level or joint level. For ROI analysis, inter-reader ICC for uric acid content was 0.73 in people level and 0.69 in joint level. Intra-reader ICC for uric acid content was 0.75 in people level and 0.71 in joint level.

**Figure 4:**
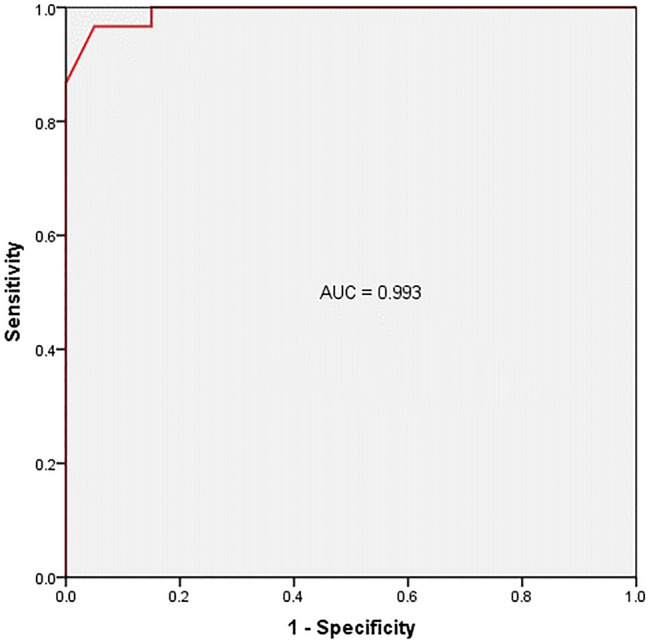
ROC analysis of MLLSV for discriminating gout patients from healthy volunteers. AUC is 0.993.

MLLSV of gout group was significantly higher than that of volunteer group (1373.3 ± 23.0 mg/cm^3^ vs. 1315.4 ± 20.7 mg/cm^3^, *p* = 0.000). The AUC of MLLSV was 0.993 in discriminating gout patient from volunteer (Fig. 4). The optimal cutoff of 1342 mg/cm^3^ corresponds with the greatest Youden-Index. When using it, the sensitivity and specificity of MLLSV in identification of gout were 96.7% and 95% respectively. (Table 1). When using the cutoff of 1332 mg/cm^3^, the sensitivity and specificity of MLLSV in identification of gout were 100% and 85% respectively. When using the cutoff of 1347 mg/cm^3^, the sensitivity and specificity of MLLSV in identification of gout were 86.7% and 100% respectively. ROI-obtained uric acid content of gout group was significantly higher than that of volunteer group (1163.7 ± 45.4 mg/cm^3^ vs. 1145.7 ± 35.5 mg/cm^3^, *p* = 0.04). The AUC of uric acid content was 0.714, significantly lower than that of MLLSV (*p* = 0.024).

**Table 1:** Comparison of MLLSV between gout group and volunteer group.

	Gout group (mg/cm^3^)	Volunteer group (mg/cm^3^)	*p* value	AUC
Patient	1372.3 ± 23.0	1315.4 ± 20.7	0.000	0.993
Ankle joint	1332.0 ± 33.5	1292.0 ± 26.7	0.000	0.823
Tarsal joint	1326.7 ± 29.8	1299.4 ± 19.5	0.000	0.786
First MTP joint	1309.4 ± 30.4	1273.4 ± 21.7	0.000	0.838

**Figure 5:**
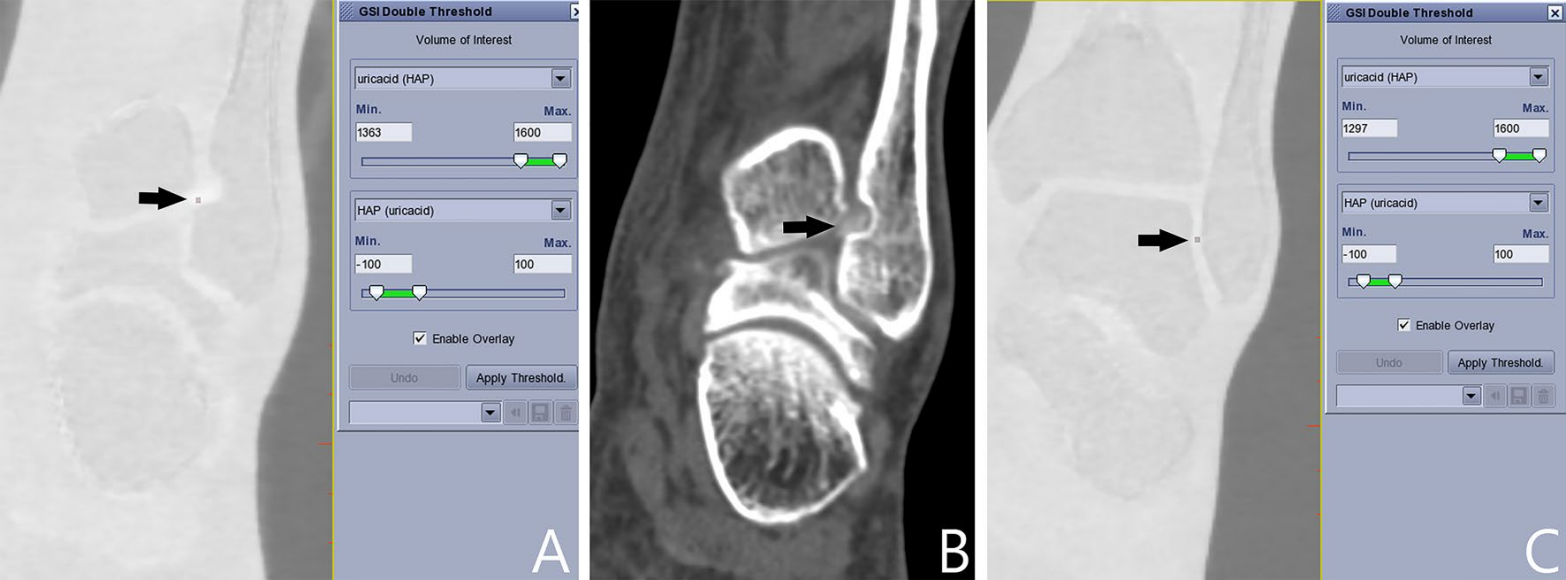
Comparison of MLLSV in ankle joint. MLLSV [arrow in (**A**)] in left ankle joint is 1363 mg/cm^3^ for a 45 years old male gout patient with left ankle pain. There is a tophus [arrow in (**B**)] in the region stained in (**A**). MLLSV [arrow in (**C**)] in left ankle joint is 1297 mg/cm^3^ for a 47 years old male healthy volunteer. MLLSV in gouty joint is obviously higher than that in the normal joint.

MLLSV was significantly higher in ankle joint, tarsal joint, and first MTP joint of gout patients versus volunteers (respectively, 1332.0 ± 33.5 mg/cm^3^ vs. 1292.0 ± 26.7 mg/cm^3^, *p* = 0.000; 1326.7 ± 29.8 mg/cm^3^ vs. 1299.4 ± 19.5 mg/cm^3^, *p* = 0.000; 1309.4 ± 30.4 mg/cm^3^ vs. 1273.4 ± 21.7 mg/cm^3^, *p* = 0.000). (Table 1). Figure 5 shows an example of MLLSV in gouty joint higher than that in the normal joint. ROI-obtained uric acid content was significantly higher in ankle joint and first MTP joint of gout patients versus volunteers (respectively, 1165.3 ± 41.9 mg/cm^3^ vs. 1147.8 ± 34.6 mg/cm^3^, *p* = 0.03; 1157.1 ± 37.4 mg/cm^3^ vs. 1142.9 ± 32.5 mg/cm^3^, *p* = 0.04.) The uric acid content AUC was 0.692 and 0.673 respectively for ankle joint and first MTP joint. ROI-obtained acid content was not significantly different in tarsal joint of gout patients versus volunteers (1154.0 ± 55.5 mg/cm^3^ vs. 1145.4 ± 47.5 mg/cm^3^, *p* = 0.08).

## Discussion

The current study investigated a novel parameter derived from post-processing procedure of DECT. The most important findings were as follows: (1) gout patients and volunteers differed significantly in MLLSV; (2) measurement reproducibility was excellent for MLLSV; (3) MLLSV was highly accurate in identification of gout.

**Figure 6:**
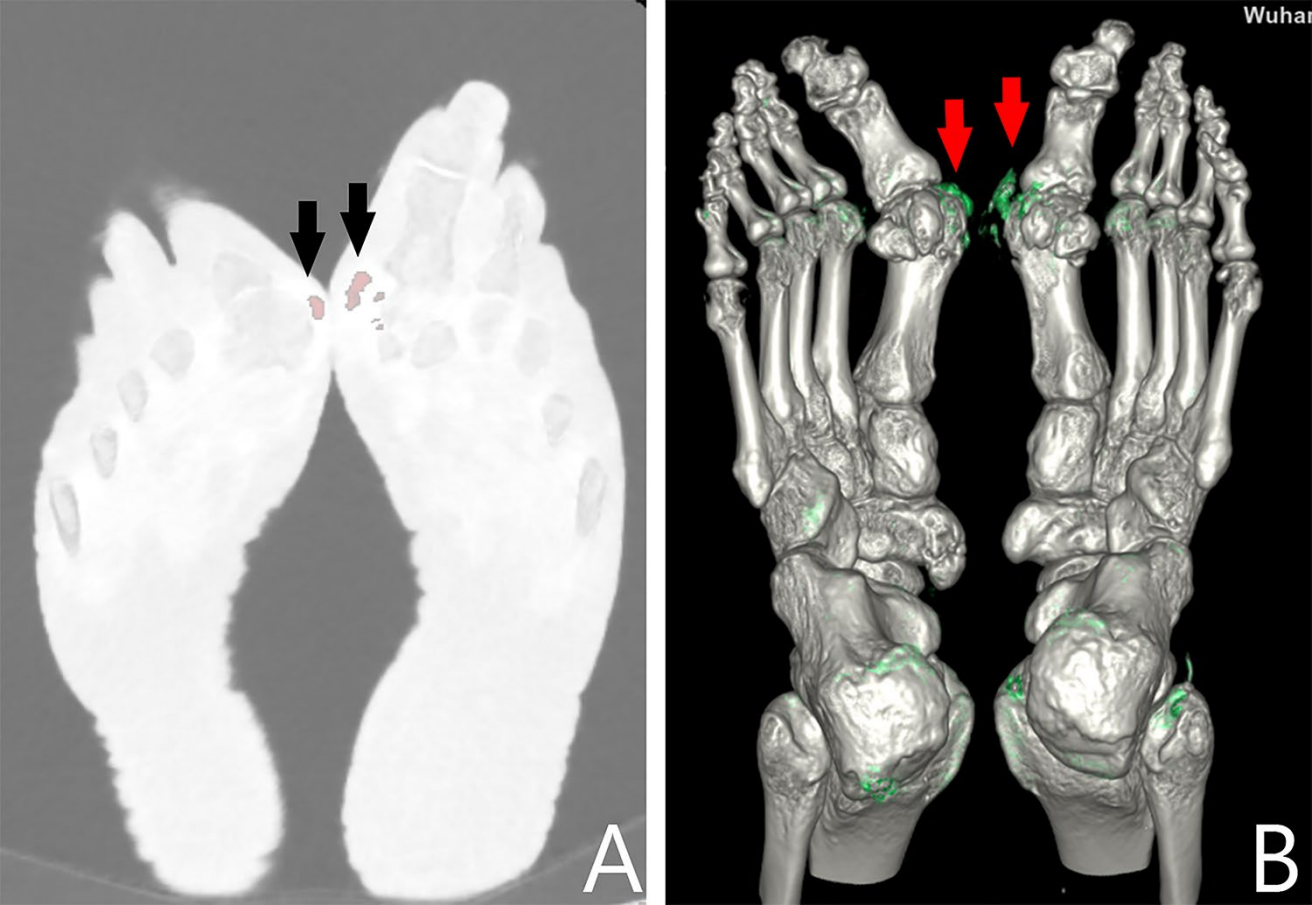
A 73 years old male patient has a gout duration of 10 years. Multiple stained sites [(**A**), arrows] can be seen in bilateral first MTP joints on the uric acid (hydroxyapatite) map. The stained sites [(**B**), arrows] can also be observed in three-dimensional volume rendering fusion map.

Either dual source or rapid-kV-switching DECT can reveal areas with higher contents of uric acid. The sites rich in uric acid are generally stained in processed images. For example, tophi are always stained red in the current study (Figs. 2B, 3). The tophi were stained because of higher uric acid level than the lower limit of detection window. The stained sites can also be observed in three-dimensional volume rendering fusion map (Fig. 6). The stained method thus helps to determine the distribution and size of tophi.

With the increase of lower limit of detection window, the number of stained sites decreased gradually, until to zero. MLLSV was thus developed by us, corresponding with the lower limit value allowing for only one stained site visible. If the lower limit of detection window is set higher than MLLSV, no stained sites will be displayed. MLLSV is thus actually the maximum value of uric acid content. We observed MLLSV varied from patient to patient, and felt it useful in identifying gout. However, there is no publications reporting the utilize of MLLSV in the diagnosis of gout. We thus obtained MLLSV for a cohort of 50 cases, and found MLLSV in gout group was significantly higher than that of volunteer group, either in people level or joint level. MLLSV also had excellent inter-reader and intra-reader agreement. In fact, MLLSV is the same for any operators, at any time. This stability is very important for either radiologists or physicians.

We found gout patients and normal people differed in MLLSV, which closely correlated with the difference of uric acid content between the two groups. Our ROI analysis did find grout group and volunteer group differed in mean content of uric acid. However, ROI-obtained uric acid value provided inadequate AUC in the study. The reasons may be as follows: (1) inter-reader or intra-reader agreement was not excellent due to ROI placement variance; (2) ROI included many tissues of normal uric acid content; (3) mean value of ROI is less than that of maximum value within ROI. In contrast, MLLSV overcame the limitations above. MLLSV can be considered as the maximum of uric acid content, which is directly caused by the gout. MLLSV performed better than ROI in identifying gout, indicating that maximum value is more suitable than mean value in diagnosing gout.

This study has limitations. First of all, invasive synovial fluid aspiration was not used as the reference standard. Gout was clinically confirmed in the current study, which seemed an imperfect standard. However, all gout patients had tophi, which increased the confidence of gout diagnosis. Second, the sample size of the study is small to moderate, especially for normal people. Due to the ionizing radiation of CT, it is difficult for us to collect volunteers. Fortunately, we scanned both sides of patient limb, doubling sample size. Ankle joint, tarsal joint, and first MTP joint were separately analyzed, also increasing sample size. In further study, we will collect more patients and volunteers. Third, we only included gout patients with visible tophi, as invasive method was unavailable to confirm the presence of uric acid crystallization. Gout patients without tophi were totally excluded in the study, but should be focused in future study.

In conclusion, MLLSV derived from post-processing procedure of rapid-kV-switching DECT is useful in discriminating gout patients from healthy people, and is a promising novel parameter for identification of gout.

## Data Availability

After publication, the data will be made available to others on reasonable requests to the corresponding author.
